# A New Inflammatory Marker: Elevated Monocyte to HDL Cholesterol Ratio Associated with Smoking

**DOI:** 10.3390/jcm7040076

**Published:** 2018-04-10

**Authors:** Mücahid Yılmaz, Hidayet Kayançiçek

**Affiliations:** 1 Department of Cardiology, Elazığ Education and Research Hospital, Elazığ 23200, Turkey; 2 Department of Cardiology, Elazığ Medical Park Hospital (Affiliated to Istinye University), Elazığ 23040, Turkey; dr.hidayet@hotmail.com

**Keywords:** smoking, monocyte to high-density lipoprotein cholesterol, inflammation

## Abstract

Objectives: The adverse effects of smoking in various pathologies are mediated by its effects on the inflammatory system. The monocyte to high-density lipoprotein cholesterol (HDL-C) ratio (MHR) has recently emerged as an indicator of inflammation. We aimed to investigate the relationship between MHR and cigarette smoking. Patients and Methods: Three hundred and ninety seven consecutive participants who smoke and 515 healthy subjects with no history of smoking enrolled in the study. Complete blood count parameters and lipid profile were analyzed in all study participants. Smoking habits were calculated as pack.years and number of cigarettes smoked per day. Results: MHR levels were significantly higher in smokers compared to non-smokers (respectively, 15.71 (12.02–20.00) and 11.17 (8.50–14.16), *p* < 0.0001)). Pearson’s correlation analysis revealed a weak but positive correlation between pack.year and MHR in the smokers group, and there was a moderate positive correlation between the number of cigarettes smoked daily and MHR in the group. In receiver operating characteristics (ROC) analyses, it was determined that a MHR value >13.00 measured in smoker participants at application had a predictive specificity of 66.6% and sensitivity of 70.0% for smoking (area under the curve [AUC] 0.729, 95% CI 0.696, 0.762; *p* < 0.0001). Conclusions: Elevated MHR is associated with cigarette smoking and may be a useful indicator of a systemic inflammatory response in smokers. Smoker participants who have high MHR levels can easily be identified during routine complete blood count (CBC) analysis and could possibly benefit from preventive treatment.

## 1. Introduction

Smoking has been causally related to several diseases, primarily those affecting the pulmonary and cardiovascular systems, including cancer, coronary heart disease and chronic obstructive pulmonary disease [1]. The World Health Organization has proposed that smoking is the single most important preventable health risk in the world [2]. Despite warnings about the health hazards of cigarette smoking, the prevalence of smoking remains high in most countries, thereby remaining a major public health concern [3]. The effects of cigarette smoking (CS) on human health have been extensively investigated at the organ, cellular, and molecular levels. Cigarette smoking has been linked to perturbations in many molecular pathways, including oxidative stress and immune response [4]. Several toxins present in CS have immunomodulatory effects. CS also contains trace amounts of microbial cell components, including bacterial lipopolysaccharides. These and other CS constituents induce chronic inflammation at mucosal surfaces and modify host responses to exogenous antigens [5].

Monocytes and macrophages are the most abundant cells that secrete proinflammatory and prooxidant cytokines as part of inflammatory reactions [6]. Moreover, it was demonstrated that high-density lipoproteins (HDL) protected endothelial cells against the noxious effects of low-density proteins (LDL) and prevented the oxidation of the LDL molecules. Therefore, it was believed that HDL had both anti-inflammatory and anti-oxidant effects [7]. In recent studies, the ratio of the monocyte count to the HDL cholesterol level (MHR) was defined as an easy calculable cardiovascular prognostic marker indicating the extent of inflammation and oxidative stress [7,8,9].

Because both inflammation and oxidative stress are the main problems of atherosclerosis caused by smoking, we hypothesized that higher MHR may be associated with the presence of smoking compared to non-smoking. Therefore, we aimed to investigate the relationship between MHR and cigarette smoking.

## 2. Materials and Methods

Three hundred and ninety seven consecutive participants (female: 139) with current smoking and five hundred and fifteen age-matched healthy participants (female: 199) with no history of smoking, who were admitted to the cardiology clinics of Elazığ Education and Research Hospital, Elazığ, Turkey, between November 2016 and January 2018, were included in this prospective study. All participants were between 17 and 75 years old and had no cardiac systemic disease or atherosclerotic risk factors (except from hyperlipidaemia). This was determined by transthoracic echocardiography (TTE) or an exercise stress test. The study was performed in accordance with the Helsinki principles and approved by the local university ethics committee.

Participants who smoked one or more cigarettes per day were accepted as smokers. Smoking characteristics such as the number of cigarettes smoked daily and the number of pack years of smoking, which represents a combined measure of dose and duration of smoking, were also evaluated. Pack.years was calculated as number of cigarettes smoked per day × number of years smoked/20.

The blood pressure records of the participants were noted. The participants having a systolic blood pressure ≥140 mmHg and/or a diastolic blood pressure ≥90 mmHg and those taking antihypertensive drugs were accepted as hypertensive.

The participants using oral antidiabetic drugs or insulin or having a measurement of fasting blood glucose level ≥126 mg/dL were accepted as diabetic.

### 2.1. Exclusion Criteria

Those excluded from the test were patients with the presence of chronic diseases, such as diabetes mellitus, hypertension, coronary artery disease, heart failure, chronic lung disease, connective tissue disease, chronic kidney disease, metabolic syndrome, thyroid dysfunction, use of non-steroidal anti-inflammatory drugs (NSAIDs) in the previous week, steroid use in the previous 6 months (including steroid creams), upper respiratory tract infection within the last 3 weeks, pregnant women, anaemia, leucocytosis, leukopenia or any other haematological, biochemical or serological abnormalities, participants with routine alcohol intake, marijuana, and use other tobacco products, and ex-smokers.

### 2.2. Echocardiography

Transthoracic echocardiography was performed using a Vivid 5 instrument (GE Medical Systems, Milwaukee, WI, USA), with a 2.5 MHz transducer and harmonic imaging according to the recommendations of the American Society of Echocardiography [10]. Left ventricular systolic and diastolic diameters were measured by M-mode echocardiography. The left ventricular ejection fraction was assessed using the Teichholz method [10].

### 2.3. Exercise Stress Test

The stress test was performed using the Bruce or modified Bruce treadmill protocols with a Cardiosis TEPA Exercise Stress Test device (TEPA Medicaland Electronic Products Industry and Trade Company, Ankara, Turkey), which are non-invasive measures of functional capacity and exercise tolerance in individuals with suspected cardiovascular disorders [11].

### 2.4. Laboratory Measurements

All blood samples (6 mL for full biochemistry, 5 mL for complete blood count) were obtained from the ante-cubital vein after 12 h of fasting. Samples were drawn into vacuum tubes containing 15% K3 ethylene diamine tetra acetic acid (EDTA)-anticoagulation tubes (Sarstedt, Essen, Belgium) and analysed. Complete blood count (CBC) parameters were assessed using a Sysmex XN-1000 haematology analyser (Sysmex Europe GmbH, Sysmex Corporation, Hamburg, Germany) according to the manufacturer’s instructions. Glucose, urea, creatinine, total cholesterol, triglycerides, high-density lipoprotein cholesterol (HDL-C) and low-density lipoprotein cholesterol (LDL) levels were measured with a Cobas^®^8000 (Roche Diagnostics International Ltd., Rotkreuz, Switzerland) auto-analyser device using the chemiluminescence method.

### 2.5. Statistical Evaluation

Statistical analyses were performed using SPSS software, version 16.0 (SPSS Inc., Chicago, IL, USA) for Windows. Continuous variables were expressed as mean standard deviations, and categorical variables were expressed as counts and percentages. The Student *t*-test and Mann–Whitney *U* test were used to compare groups for continuous variables, and the chi-square test was used for categorical variables. Normality of the distribution of the continuous variables was evaluated using the Kolmogorov–Smirnov test. Age, triglycerides, total cholesterol, LDL cholesterol, HDL cholesterol, monocytes, MHR, BMI (body mass index) and platelet counts did not show normal distribution and the Mann–Whitney *U* test was used to compare these parameters. Correlation analyses were performed using Pearson’s correlation test. Receiver-operating characteristic (ROC) analysis was performed for predicting optimal cut-off values of MHR in the presence of smoking. All *p*-values were two-tailed, and values <0.05 were considered to indicate statistical significance.

## 3. Results

**Figure 1:**
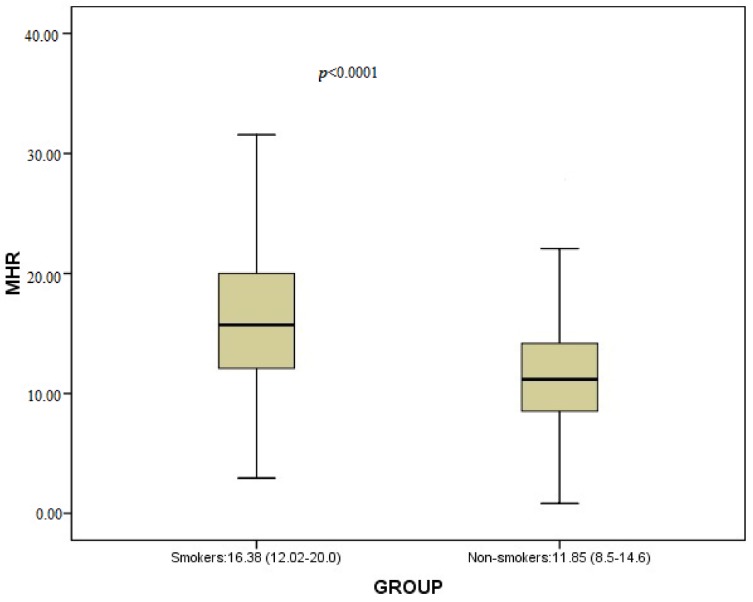
Comparison of MHR between smokers and non-smokers.

**Figure 2:**
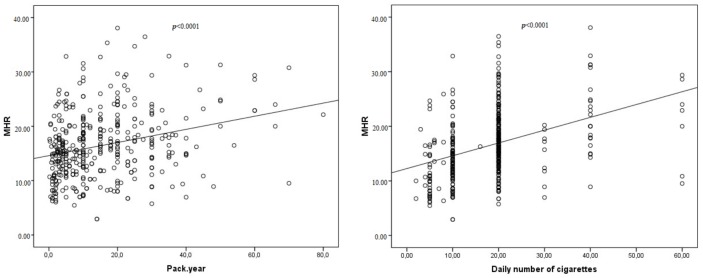
Correlations between MHR and duration of smoking (**A**) and number of cigarettes per day (**B**) in smokers.

The study included 912 consecutive healthy participants. There were 397 (female: 139) smokers and 515 (female: 199) non-smokers. It was observed that MHR values for the smoker group were significantly higher than those of the non-smoker group (respectively, 15.71 (12.02–20) and 11.17 (8.50–14.16), *p* < 0.0001) (Table 1, Figure 1)). Triglycerides, high-density lipoprotein cholesterol (HDL-C), WBC (white blood cell), monocytes, haematocrit and haemoglobin values for the smoker group were significantly higher than those of the non-smoker group (Table 1). BMI (body mass index) was significantly lower in the smoker group than the non-smoker group (Table 1). The present study also displayed increased HDL levels and decreased MHR values in females in both groups (Table 2). While there was a weak positive correlation between pack.year and MHR, there was a moderate positive correlation between the number of cigarettes smoked daily and MHR in the smoker group (Table 3 and Table 4, Figure 2). Although there were no statistically significant differences between the two groups regarding total cholesterol, for low-density lipoprotein cholesterol (LDL-C), there were positive correlations between these parameters and pack, year–the number of cigarettes smoked daily in smoker group (Table 3 and Table 4, Figure 2).

**Figure 3:**
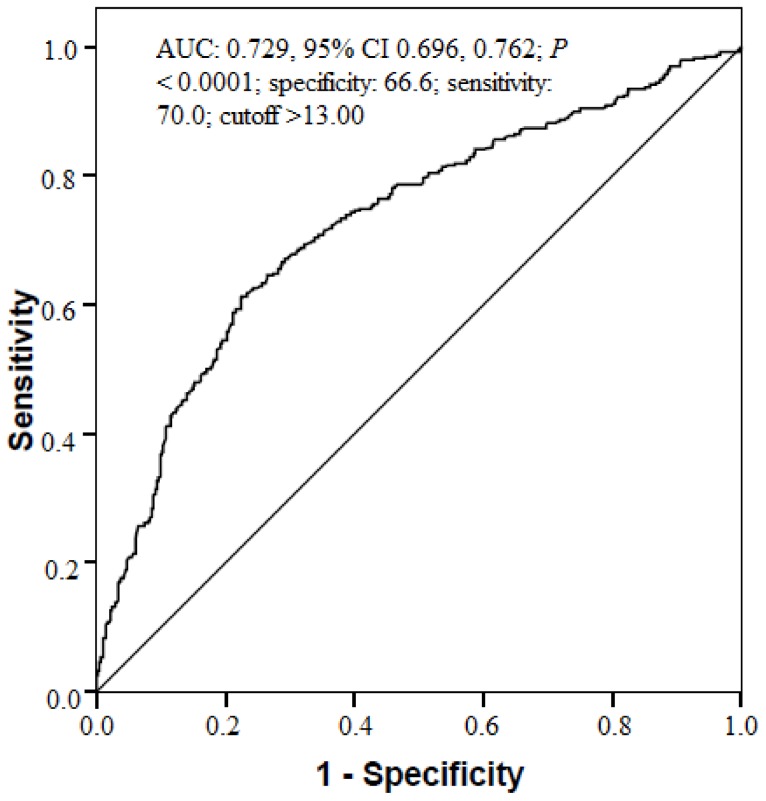
MHR receiver-operating characteristic (ROC) analysis between smokers and non-smokers. AUC, area under the curve; CI, confidence interval; MHR, Monocyte to HDL-C ratio; ROC, receiver operating characteristics.

The ROC curve analysis demonstrated that the specificity of an MHR value >13.00 (calculated prior to taking anamnesis) in predicting smoking cases was 66.2%, and the sensitivity was 70.0% (area under the curve [AUC] 0.729, 95% CI 0.696, 0.762; *p* < 0.0001) (Figure 3).

There were no statistically significant differences between the smoker group and non-smoker group in terms of hyperlipidaemia, age and other investigated laboratory parameters (Table 1).

## 4. Discussion

In the present study, we found that MHR, triglycerides, high-density lipoprotein cholesterol (HDL-C), WBC (white blood cell), monocytes, haematocrit and haemoglobin values were significantly higher in the smoker group than in the non-smoker group. We also found BMI (body mass index) values of the smokers group was significantly lower than non-smokers group (Table 1). In addition, there were positive correlations between pack.year–the number of cigarettes smoked daily and the plasma level of MHR, total cholesterol, triglycerides, LDL-C, HDL-C, and monocytes (Table 3 and Table 4, Figure 2). The study also showed that there was a stronger correlation between MHR and the number of cigarettes smoked daily when compared to correlation between pack.year and MHR in the smokers group (Table 3 and Table 4, Figure 2). As presented in Figure 2, MHR had a moderate and significant degree of correlation with amount of smoking and a weak but still significant correlation with duration of smoking (r = 0.379, *p* < 0.0001; r = 0.273, *p* < 0.0001, respectively). These results indicate that elevated serum WBC, monocytes and MHR levels may be associated with ongoing inflammation in the pathophysiology of cigarette smoking. Furthermore, we can say elevated levels of MHR are more associated with amount of daily smoking than duration of smoking (pack.year). The results also indicated that elevated haemoglobin and haematocrit consentrations and dyslipidaemia observed in smokers may be associated with CS.

Triglyceride and HDL-C analysis in our study groups were significantly different between the two groups. These findings are consistent with previous findings that showed higher serum levels of triglyceride concentrations and lower plasma concentrations of HDL-C in smokers [12].There were no differences between the group in terms of total cholesterol and LDL-C analysis, but correlation analysis revealed a positive relationship between two parameters and pack.year–the number of cigarettes smoked daily. BMI values of the non-smokers group was significantly higher than the smokers group in the present study. This situation may be responsible for the results of the statistical analysis. Secondly, there were no differences between the two groups in terms of hyperlipidaemic participants included in our study. This situation may be another reason for the lipid profile results. Finally, dietary differences between smokers and non-smokers in our study might have caused these results.

There is much evidence to show that chronic systemic inflammation has a major role in the development of atherosclerosis [13,14,15]. The effects of the CSon systemic inflammatory response is well defined in several studies [16,17,18]. In a multi-ethnic cohort study, both former and current CS were found to be independently associated with markers of inflammation and subclinical atherosclerosis. In this study, the associations were found to be stronger for current smokers than for former smokers [19]. According to several recent studies, exposure to CS impairs the functional structure of endothelial cells. Nicotine and the increased oxidative stress generated from smoking induce vascular endothelial dysfunction via the inhibition of endothelial nitric oxide synthase and decreasing generation of nitric oxide [20,21]. Furthermore, nicotine increases the expression of adhesion molecules in endothelial cells, such as E-selectin and intracellular adhesion molecular 1, because of enhanced attachment and transmigration of monocytes to the vessel wall [22]. It is evident that these results suggest that smoking is an established risk factor for atherosclerosis through several underlying pathways [23].

Monocytes are distinct types of leukocytes which have a key role in inflammation and the atherosclerosis process [6]. Activated monocytes interact with damaged or activated endothelium, which results in the overexpression of proinflammatory cytokines/adhesion molecules, including monocyte chemotactic protein 1 ligand, vascular cell adhesion molecule 1 and intercellular adhesion molecule 1. Thereafter, monocytes differentiate into the macrophages that ingest oxidized LDL-C and form dangerous foamy cells [24]. In another study, the count of circulating monocytes was found to be a predictor for new plaque development as well [25]. However, HDL-C features anti-inflammatory, anti-oxidant and anti-thrombotic effects [24,26,27]. HDL-C can prevent inflammatory responses by acting directly on monocytes. Recent studies indicate the role of HDL-C in modulating monocyte activation, adhesion and in controlling the proliferation of progenitor cells that differentiate to monocytes. HDL-C also prohibits oxidation of LDL-C in addition to inhibition of macrophage migration. It also removes oxidized LDL-C from foamy cells [26,27,28,29,30]. Therefore, monocytes show a pro-inflammatory effect, but HDL-C functions as a reversal factor during this process.

It has been suggested that MHR has a relationship with systemic inflammation and endothelial dysfunction, and it is accepted as a newly recognised inflammation-based diagnostic and prognostic marker in cardiovascular diseases [31,32,33,34]. Recently, Acikgoz et al. assessed endothelial function using flow- and nitro-glycerine-mediated dilatation techniques and the calculation of MHR. The study reported that there was a strong inverse correlation between MHR and flow-mediated dilatation. Therefore, elevated MHR may be a useful marker reflecting impaired endothelial function and systemic inflammation [35].

The relationship between smoking, systemic inflammatory response, vascular endothelial injury and atherosclerosis has been well defined both in the past and more recently [20,21,22,36]. In light of the information given above, we can hypothesize that MHR reflected systemic inflammation and endothelial dysfunction expected from smoking status. In the present study, we found that MHR levels were significantly higher in smokers than in non-smokers, and there was a relationship between MHR and pack.year–the number of cigarettes smoked daily. Therefore, MHR may be used as a surrogate marker of inflammation and endothelial dysfunction in smokers. When laboratory data are seperated by gender, females had higher HDL-C levels and lower levels of MHR than males. These findings suggest that males are more likely to develop vascular endothelial dysfunction and atherosclerosis than females. It is of course likely that we need to assess vascular endothelial dysfunction with an invasive method, such as the flow-mediated dilatation technique, in addition to MHR calculation. Even so, MHR calculation may provide considerable information for the examination of smokers in an outpatient setting.

## 5. Conclusions

MHR is a simple, easy, cost-effective tool that should be used for predicting the systemic inflammatory response and possible endothelial dysfunction in smoker cases. Cases with high MHR levels can easily be identified during routine complete blood count (CBC) analysis and could possibly benefit from preventive treatment. Therefore, more attention should be given to these indices in the examination of a smoker case.

## 6. Limitations

We could not control the exposure to second- and third-hand smoking. This problem may lead to some errors in the interpretation of the results.

## Figures and Tables

**Table 1:** Inter-group comparison of demographic and laboratory data.

	Smokers (397)	Non-Smokers (515)	*p* Value
Gender (Male/Female)	258/139	316/199	0.26
Age (year)	37 (26.0–47.0)	34 (24.0–47.0)	0.10 ^#^
Hyperlipidemia n (%)	78 (19.6)	82 (15.9)	0.14
Triglycerides (mg/dL)	132 (94.75–186.0)	102 (72.0–147.0)	<0.0001 ^#^
Total cholesterol (mg/dL)	177 (150.0–202.5)	172 (149.0–198.0)	0.33 ^#^
Low-density lipoprotein cholesterol (mg/dL)	104.0 (80.39–123.0)	99 (78.0–120.0)	0.07 ^#^
High-density lipoprotein (HDL) cholesterol (mg/dL)	41 (35–49)	50 (43–58)	<0.0001 ^#^
Monocytes (×10^3^/mm^3^)	0.65 (0.53–0.78)	0.56 (0.46–0.67)	<0.0001 ^#^
Monocyte to HDL-C ratio (MHR)	15.71 (12.02–20)	11.17 (8.50–14.16)	<0.0001^#^
BMI (body mass index)	25.40 (24.28–27.35)	26.26 (25.36–27.21)	<0.0001^#^
Platelet (×10^3^/mm^3^)	262 (228.0–301.0)	270 (230.0–313.0)	0.09 ^#^
White blood cell (×10^3^/mm^3^)	8.01 ± 1.97	7.29 ± 1.69	<0.0001
Glucose (mg/dL)	94.73 ± 18.66	94.25 ± 15.91	0.68
Sodium (mmol/L)	139.89 ± 3.28	140.09 ± 2.77	0.34
Potassium (mmol/L)	4.27 ± 0.41	4.25 ± 0.47	0.50
Calcium (mg/dL)	9.32 ± 0.53	9.37 ± 0.59	0.20
Urea (mg/dL)	28.14 ± 7.94	28.21 ± 8.65	0.90
Creatinine (mg/dL)	0.60 ± 0.16	0.60 ± 0.17	0.91
Hematocrit (%)	43.88 ± 3.21	41.88 ± 2.98	<0.0001
Hemoglobin (g/dL)	14.64 ± 1.09	13.63 ± 1.03	<0.0001

**^#^** Normality of the distribution was evaluated by the Kolmogorov–Smirnov test and the Mann–Whitney *U* test applied to compare for continuous variables.

**Table 2:** Comparison of demographic and laboratory data between females and males.

	Smokers (397)		Non-Smokers (515)		*P_1_*	*P_2_*
	Males (258)	Females (139)	Males (316)	Females (199)		
Age (year)	33 (24–46)	40 (35–47)	33.5 (23–49.75)	36 (26–46)	<0.0001 **^#^**	0.67 **^#^**
Hyperlipidemia n (%)	48 (18.6)	30 (21.6)	43 (13.6)	39 (19.6)	0.47	0.07
Triglycerides (mg/dL)	136.5 (96.75–190)	130 (84.10–168.0)	107 (75–150)	95 (66–140)	0.07 **^#^**	0.07 **^#^**
Total cholesterol (mg/dL)	172.95 ± 39.66	187.68 ± 39.12	173.5 (150–198)	171 (147–200)	<0.0001	0.7 **^#^**
Low density lipoprotein cholesterol (mg/dL)	100.73 ± 32.54	110.20 ± 35.72	101 (82–120)	91 (73.64–114)	0.01	0.003 **^#^**
HDL cholesterol (mg/dL)	40.08 ± 9.41	49.48 ± 13.41	46.75 (41–56)	55 (48–64)	<0.0001	<0.0001 **^#^**
Monocytes (×10^3^/mm^3^)	0.69 ± 0.18	0.59 ± 0.16	0.58 (0.48–0.69)	0.52 (0.43–0.63)	<0.0001	<0.0001 **^#^**
MHR	18.10 ± 5.77	13.19 ± 5.60	12.32 (9.51–15.68)	9.30 (7.76–12.07)	<0.0001	<0.0001 **^#^**
BMI (Body mass index)	25.45 ± 2.20	25.66 ± 2.16	26.36 (25.55–27.42)	26.10 (24.97–27.05)	0.35	0.011 **^#^**

P_1_: between males and females in smokers; P_2_: between males and females in non-smokers; **^#^** Normality of the distribution was evaluated by the Kolmogorov–Smirnov test and the Mann–Whitney *U* test applied to compare for continuous variables.

**Table 3:** Pearson’s correlation analysis between smoking as pack.year, MHR and blood lipid levels.

Variable	Pack.Year	
	r	*p*
MHR	0.273	<0.0001
Monocytes (×10^3^/mm^3^)	0.205	<0.0001
HDL cholesterol (mg/dL)	−0.155	0.002
Triglycerides (mg/dL)	0.242	<0.0001
Total cholesterol (mg/dL)	0.201	<0.0001
Low density lipoprotein cholesterol (mg/dL)	0.200	<0.0001

**Table 4:** Pearson’s correlation analysis between the number of cigarettes smoked daily, MHR and blood lipid levels.

Variable	The Number of Cigarettes Smoked Daily	
	r	*p*
MHR	0.379	<0.0001
Monocytes (×10^3^/mm^3^)	0.321	<0.0001
HDL cholesterol (mg/dL)	−0.229	0.002
Triglycerides (mg/dL)	0.203	<0.0001
Total cholesterol (mg/dL)	0.112	0.025
Low-density lipoprotein cholesterol (mg/dL)	0.146	0.004

## Abbreviations

AUC	area under the curve
CI	confidence interval
MHR	monocyte to HDL-C ratio
ROC	receiver operating characteristics
